# Modifiable determinants for the success or failure of inter-physician collaboration in group practices in Germany - a qualitative study

**DOI:** 10.1186/s12875-020-01349-w

**Published:** 2020-12-21

**Authors:** Lisa-Marie Weinmayr, Ruben Zwierlein, Jost Steinhäuser

**Affiliations:** Institute of Family Medicine, University Hospital Schleswig-Holstein, Campus Luebeck, Ratzeburger Allee 160, 23538 Luebeck, Germany

**Keywords:** Inter-physician collaboration, Entrepreneurship skills, Strategies against physician shortage, Private practice, Group practice

## Abstract

**Background:**

A growing demand for physicians exists worldwide. Due to political changes, economic incentives and new workplace expectations a trend from single-handed practices to group practices has been noticed in many countries over the last years. In view of this background, our study aimed to identify determinants for success or failure of inter-physician collaboration in order to positively influence future collaboration in anticipation of the important role group practices may play in future health care delivery.

**Methods:**

We chose a qualitative study design, using semi-structured phone interviews to collect data from physicians and non-physician consultants with experience in inter-physician collaboration that were analysed using content analysis. Eleven physicians with experience in collaborative working and fourteen non-physician consultants specializing in advice to health care professionals participated.

**Results:**

Education in entrepreneurial skills as well as implementation of good practice management in preparation for collaboration are crucial modifiable facilitators for successful inter-physician collaboration. Furthermore, open communication and realistic insight into the mode of acquaintance, moral concept and degree of specialisation of the colleagues involved play major roles for the success of inter-physician collaboration in group practices.

**Conclusions:**

There are several underlying themes beyond clinical expertise concerning success or failure of group practices. To influence future collaboration positively, it is important to focus on management and communication skills as well as to address basic understanding of economics.

## Background

In many countries, the demand for physicians is growing and therefore concerns about local shortages exist, especially for general practitioners (GPs) and in rural areas [1, 2].

Primary care services constitute the essential part of outpatient care that is delivered in private practices in many countries, as opposed to publicly owned practices or health care centres, depending on the type of health care system [3]. In countries with a national health care system, specialists usually do not work in private practices, whereas in countries with a social security-based health system, specialists work mainly in a private setting. In fourteen European countries, including France and Germany, GPs are mainly self-employed. This means they receive payment via the framework of the respective health care system but own their practice and are responsible for investments and staffing [4].

In recent years there has been a shift from single-handed practices to group practices in outpatient care. In nine OECD countries group practices are already the predominant form of health care delivery. In direct comparison to single-handed practices, delivering health care in group practices performed better in terms of quality of infrastructure and workload [5]. Studies suggested that physicians in group practices have better prescription appropriateness, are more likely to treat patients in accordance with guidelines and have better equipment [6]. Regarding patient-centred outcomes, a positive correlation has been shown with patient satisfaction and accessibility [5–7].

Additionally, “colleagues” are an important aspect of job satisfaction. The opportunity for collegial exchange can be a motivation for working in a group practice [8–11].

While one factor of this trend towards group practices are political changes in the delivery of (primary) care and economic incentives, another important factor are the expectations young physicians have of the workplace [3, 12–14]. Especially the latter suggests that collaboration between physicians will play a crucial role in the delivery of health care in the future.

Germany has a social security-based health care system with a mandatory health insurance and free choice of doctor for the patients with no gate keeping system [15]. Treatment of patients takes place in hospitals and in surgery-based practices, which provide primary as well as secondary ambulatory care. Office-based doctors work as self-employed in their own practices and need to be registered within the Association of Statutory Health Insurance Physicians (Kassenärztliche Vereinigung, KV) in order to be part of the statutory health ambulatory medical care system [16, 17]. Physicians generate their income mainly through claims against patients with statutory health insurance. Claims of patients with private health insurance and individual health services are additional income sources [18]. As a result of not acquiring entrepreneurial skills during under- or postgraduate training, newly qualified physician depend on various professions (e.g. lawyers, tax consultants) during the process of setting up their doctor-office [19].

Overall, with a 55% share, single-handed practices are still the predominant practice form in Germany. However, there is a trend towards grouping together [20, 21]. The term collaboration refers to a number of association models for physicians that collaborate in private practice, including group practices, ambulatory healthcare centres and other models. These association models differ in terms of liability, allocation of profits and temporal representation [22]. Collaborations can include physicians of the same area of expertise as well as an interdisciplinary collaboration, e.g. between specialists and GPs; however, collaboration between physicians of different specialisations are less common [21].

More than a third of the practicing GPs in Germany are 60 years or older, which means that these will look for a successor in the next ten years [23]. Consequently, more than 80% of the vacancies for office-based physicians are scheduled for GPs according to the demand-oriented planning of the KV [24].

Considering the statistics for German physicians’ and practice type trends referred to earlier as well as the workplace perceptions of newly qualified physicians, it becomes clear that inter-physician collaboration will play a major role for delivering general practice in Germany in the future [20, 21, 23–25].

A search of the literature revealed some data about collaboration between physicians in hospitals regarding costs or effectiveness of patient treatment [26, 27]. However, in spite of the significance for future health care delivery, little data on inter-physician collaboration in group practices and about how successful collaboration between physicians functions itself can be found. According to a French study analysing group practice break-ups, obstacles to the success of group practices were imbalances in the group, communication issues and different work and management styles [28].

In view of this background, our study aimed to identify determinants for success or failure of inter-physician collaboration in group practices in order to positively influence future collaboration in anticipation of the important role group practices may play in future health care delivery.

## Methods

### Study design

We chose a qualitative study design matching the explorative character of the study, using semi-structured phone interviews.

A semi-structured guide was developed based on the SPSS method (German: “sammeln, prüfen, sortieren, subsumieren”; English: “collect, test, grade, subsume”) by Helfferich [29] starting with literature research and brainstorming within the research team. The original idea for this study and important aspects for the discussion within the team derived from a meeting with mayors of a district in Germany where JS presented results of a set of studies regarding future close-to-home health care on the 15th November 2014 [30, 31].

Two interviews, one with a physician and one with a consultant, served as pilot interviews for the guide. The interview guide was finalized afterwards by LW (female doctoral thesis student) and JS (male GP); two questions were added. The final interview guide contained the following questions:

1. What is your experience with working collaboratively in a group practice?
2. In your opinion, what are the reasons why collaboration between physicians fails?
3. In your opinion, what is essential to make collaboration between physicians last?
4. Do you think there are warning signs for failing collaborative work in a group practice?
5. What advice would you give a good friend who wants to practice collaboratively in a group practice?

Most interviews were one-to-one and conducted by LW. They were carried out in a single phone session from the Institute of Family Medicine in Lübeck, Germany. Three interviews took place at a private workplace to be able to offer appointments at the participant’s convenience. In addition, each interview was subsequently discussed with JS and ideas were documented. It was permitted to adjust the order of the guideline questions to the course of the interview. The interviews audio was fully recorded and subsequently transcribed verbatim in German using pseudonyms. Each transcript was assigned a random participant number from one to 25. Transcripts were not returned to the participants for correction for reasons of feasibility. No interview was repeated but they were proofread and compared to the audio data by an experienced study nurse. As participants did not check the findings, no feedback was provided. Data collection took seven months in total and ran from summer 2015 until the beginning of 2016. Participants were not paid nor received any other inducement for their participation. The German quotes were translated into English following coding and analysis by LW and were counterchecked by an English native speaker for comprehensibility.

### Participant sampling and recruitment

Our target group were physicians with experience of working collaboratively in group practices (currently or previously) and non-physician consultants specialised in health care professions. As there are no suggestions in the literature that the success or failure of a collaboration is dependent on the specialisation of a physician, there was no focus on a certain stratification regarding specialisations in the selection of the participants.

In Germany a number of support services for physicians starting a private practice are available, including establishment guidance by the regional KV, solicitors specialising in health care law, as well as corporate, financial and tax consultants concentrating on physicians in private practices. In order to broaden the insights into collaboration, we decided to include the experiences of these occupational groups which have a consulting function but do not directly participate in collaboration itself. Further criteria for inclusion were adequate German language skills, legal age and a signed declaration of consent. We recruited participants by convenient sampling, contacting potential participants after an online search of federal Associations of Statutory Health Insurance Physiciansfor the contact persons and personal contacts to physicians. In addition, we used the snowball method to recruit participants by asking those who had agreed to participate to put other members of their profession likely to be interested in contact with our research team. All participants were contacted via email, sending them a cover letter (introducing the interviewer and the purpose of this research), study information for participants and a consent form. No relationship was not established prior to the study, however a few participants knew members of the research team from previous occasions.

### Data analysis

The analysis was conducted using an approach based on content analysis according to Mayring [32]. Individual interviews were included in the analyses and subsequently defined as the analysis unit. The qualitative content analysis and coding had the goal of arranging the large volume of text into compact, content-bearing categories. Categorization was either preformed, deductive, text-oriented, or inductive [33–35]. For this work, deductive main categories were first derived from the interview guide and assigned to the corresponding text passages. In a second step, inductive categories were successively formed from the remaining transcripts. Several text runs were necessary in each case.

First, each interview was discussed between LW and JS and analysed for new insights in order to be able to define saturation later on. After saturation with regard to content was reached, two independent researchers (LW, doctor and RZ, male medical student and Bachelor of Science in Industrial Engineering and Business Management) coded the data without software support. Twenty-five full transcripts in the form of Microsoft Word documents were determined as the raw material. The size of the sample (T = 25) was determined by saturation with regard to content after discussion of the temporary results. Each transcript was looked at individually and in context with the other transcripts. While reading the transcripts, text passages matching categories were highlighted and coded. Afterwards these passages were reduced, paraphrased and assigned into categories. Deductive categories were derived from the questions of the interview guide. The category system was complemented by inductive categories. These categories originate from the content of the material. Both coders produced drafts for a system of categories, which were discussed to create a final version according to the principle of intercoder reliability [32]. To ensure a clear delineation of the categories, the subcategories were defined in relation to their respective main category. The resulting consistent coding agenda was used to repeat the coding of all transcripts, thus ensuring the completeness of the coding guide. Finally, each subcategory was assigned an anchor quotation as a concise core statement. The participants were not involved in this process. The final version was produced in the presence of a third person (JS), an experienced qualitative researcher, who had supervised the interviewing process and took part in discussing the categories objectively. Finally, all categories were defined as modifiable or not modifiable.

An applicable quality criterion of qualitative research for this study is intersubjective traceability. This can be divided into three sub-criteria: documentation of the research process, interpretation in groups; and application of codifying procedures [36].

According to the first sub-criterion, the research process should be documented with sufficient and transparency that all research steps, including the results, will be fully comprehensible and plausible for external readers. This was achieved by comprehensively documenting the survey methods and the survey context, the transcription rules, the data and the evaluation methods. Furthermore, the researchers’ prior understanding, in this case their medical background, was explicitly communicated to the participants.

The second sub-criterion – the interpretation in groups – was achieved through the previously described coding consensus by the three researchers. Here the focus was on the clear and differentiated handling of data and their interpretation.

The application of the qualitative content analysis, already described as a codifying procedure, ensured a certain standardisation of the methodology, which allowed checking by external readers and increased transparency. Thus, the third sub-criterion was also fulfilled [36].

The COREQ (COnsolidated criteria for REporting Qualitative research) checklist provides a guideline for the transparent and comprehensive reporting of qualitative research and the items of this checklist have been considered for this work [37]. The fully completed checklist can be found in the appendix.

## Results

Overall, we interviewed twenty-five participants. On average the interviews lasted approximately twenty-five minutes. The majority of the sample was male. Eleven participants were physicians and fourteen were non-physician consultants who specialised in advising self-employed, collaborating physicians. Out of all the people contacted, most non-participants did not state a reason for their non-participation, as they did not respond at all. One person refused to participate because she felt she was “not qualified”. Table 1 gives further characteristics of the study sample and the interviews. Citations below are marked P for physician or NP for non- physician.

**Table 1:** Characteristics of the study sample (*n* = 25) and interviews

		N* (%)
Gender	Male	19 (76)
	Female	6 (24)
Profession	Solicitor	5 (20)
	Tax consultant	2 (8)
	Financial consultant	4 (16)
	Establishment advisor	3 (12)
	Physician	11 (44)
	general practitioner	6 (24)
	internal medicine	3 (12)
	dermatologist	1 (4)
	urologist	1 (4)
		Mean (min./ max.)
Age (years)		50 (25/ 65)
Interview duration (minutes)		25 (14/ 34)
Work experience (years)	All participants	22.5 (6/ 36)
	Solicitor	15 (9/ 20)
	Tax consultants	17 (15/ 18)
	Financial consultants	18 (6/ 26)
	Establishment advisors	18 (16/ 18)
	Doctors	29 (16/ 36)

*Numbers vary because of missing data (N number, min minimum, max maximum)

Table 2 shows all categories (determinants for success or failure) found within the data divided into modifiable and non-modifiable categories. In the following, we present those factors identified as modifiable for success or failure of group practices. The main categories, in view of modifiable determinants, that arose were *communication, the collaboration partner, practice management, preparation for group practice, lack of entrepreneurial skills* and *financial aspects.*

**Table 2:** System of categories: determinants for success or failure of inter-physician collaboration

	Main category	Sub-category	Anchor quotation
**Modifiable**	Communication	Professional level	*P22: Even if there are no issues I would advise everyone to set up structured meetings that ensure feedback and consultation. It doesn’t have to be every week, but once a month or quarter.* *And it should be in a casual pub atmosphere. So that people can really speak up about anything.*
		Personal level	*P4: […] everyone should clearly state their interests […] and negotiating always means that both have to compromise […] from today’s point of view, I would say spending 2000–3000 Euro for a facilitated negotiation with a professional would have been a good investment.*
	Collaboration partner	Mode of acquaintance	*NP10: […] and I notice that the best and most secure collaborations are the ones where junior doctors, who have worked in the practice join in. [...]if somebody had worked in the practice for some time already, for their specialisation or as a locum, if they know the practice [..].*
		Moral concept and values	*P4: Strong differences in moral concepts and values lead to failure, that is quite simple. […]*
		Degree of specialisation	*NP8: […] On the other hand, as soon as you are more specialized in a group practice or an association, it reduces comparability; and the less comparability the less trouble develops […]*
	Practice management	Organisation in general	*NP1: But this is always subjective and the more professional the level, meaning the more people work together, the more intense the organisational structure, maybe with a manager or as alimited company, the less relevant these elements become, and fewer will fail. So I think if you want to build a long- lasting arrangement, you have to try to raise the professional level.[…]*
		Staff	*P4: […] We treat staff very differently, which makes them insecure. This was one condition that led to the failure of our practice.[…]*
		Distribution of duties	*P4: […] in my personal opinion it’s not the money, or financial imbalance, in fact it’s the uneven distribution of administrative duties, like computer systems* etc.
		Consulting services/ Delegation	*NP12: […] and to organise the things you’re not familiar with, that’s the key solution. To have a good tax consultant, so someone is in control, to have a bank that you trust involved, these are the things that I don’t want to deal with when I’m in my practice treating patients. And I have to find somebody else to do it […]*
	Preparation for the group practice	Contractional protection	*P4: […] define target agreements and – and this is absolutely necessary – to make contracts […]*
		Lack of work experience	*P19: […]what I would advise someone above all else is: first of all, to work in a private practice, even if only for some hours or*
			*weeks, just to see the work routine. […] Because private practice is very different from working in a hospital […]*
		Time management	*NP9: […] it’s always successful if they start planning in time, sensible planning, this is a common issue, even overnight, because there is a lot of time pressure.*
		Use of consulting services	*P21: […] many see our professional body, the Association of Statutory Health Insurance Physicians almost as an enemy if you want to put it that way. This is very foolish, because it’s a very competent, powerful and supportive organisation […]*
	Financial aspects	Allocation of profits	*NP15: […] and I would say, a common core issue is the allocation of profits and losses, if someone thinks they have worked more than the other and perceives the allocation of profits, if it is 50:50, as unfair.*
		Accounting	*NP2: [...] They have an early warning system that realizes very quickly if something is going on. [...] the smaller [practices] send their billing to the KV every quarter and wait to see what will happen instead of having done in an organised fashion [...]*
		Disappointed by lack of synergies	*NP3: […] physicians expect that if they enter a collaboration, that they can basically cut their expenses in half. But it doesn’t work like that, and sometimes if expectations are too high, that can be a reason for failure, too. […]*
	Entrepreneurial skills	Lacking preparation to self- employment	*P21: […] A lot of naivety. Physicians start a private practice without any preparation. In terms of human, economic or any other form of organisational preparation. They just do it. […]*
		Underestimated complexity	*NP13: What is very important for me is entrepreneurial awareness. Many doctors stumble into a collaboration without realizing that they have equal partners, that they share an entrepreneurial position. They underestimate the decisions they have to make in important business matters, in contractual matters. […]*
		Hospital vs. private practice	*NP2: […] they don’t talk about where they are going with their practice, how to handle self-payers, how much money do I require for my personal needs, how should we handle staff issues […] They don’t know this, if they have only worked in a hospital.*
		Practice Concept	*NP11: […] I think one of the main reasons is a deficit that almost every practice has, that there is no coordinated cooperative concept among the partners. […] I believe that numbers are easy, and every economist can illustrate numbers and build a finance plan. The more difficult part is to*
			*agree on ideas for a concept like: Where do I want to go with my practice? How does it fit? What does my partner want? What do we want to offer in medical terms? And all these things […]*
**Not modifiable**	Gender specific aspects	Wish for collaboration Competitiveness	
	Willingness to cooperate	Motivation for collaboration Financial commitment Medical specialty	
	Personality	Favourable qualities Unfavourable qualities Constellation of personalities	
	Personal consideration	Aims Self-esteem and content	
	Mode of operation	Division of work Working pace Commitments outside the practice	
	Interpersonal aspects	Between doctors Working climate	
	Medical expertise	Professional consensus Degree of specialisation Liability	
	Private aspects	Interference from spouses Changed consumer behaviour Altered environment	
	External factors	Economic crisis Urban/rural differences	

### Communication

Almost all participants felt that communication is one of the most basic requirements for good collaboration and is therefore a crucial factor for the success of working collaboratively in a group practice.

They outlined the value of open communication for working collaboratively on a professional level, especially at the beginning of the exchange of expectations and the definition of common goals, as well as for interpersonal relationships in daily work. Regular meetings are the best way to ensure good communication.

P23: […] to run through all eventualities. To sort out everything you can as long as you are getting along. […] for example what happens if I die or get sick […].

Consequently, several respondents emphasised that a lack of communication and agreement can lead to failure of collaboration, especially as in a group practice all decisions have to be taken together.

*P17: […] in a group practice anything carries the potential for conflict, if you have to make decisions or arrange tasks. It’s because there are two bosses or even more and that requires close consultation among each other.*

*NP15: Everyone has their own ideas on how collaboration works and yes, that means that a lack of communication usually is a big problem.*

Another aspect is the personal level of communication: a partner should be able to clearly define their own interests but also be willing to compromise. To this end, both physician and non-physician participants reported benefits by hiring an independent facilitator or mediator to support important meetings and negotiations.

*NP6: [...] and they do this on a regular basis, hold the doctors’ meeting under the supervision of a facilitator, or mediator, because it organises the communication process, structures it, and that means even sensitive issues can be discussed easily.*

### Collaboration partner

Both groups named the physician partner as a determinant for success or failure. Sub-categories consist of *mode of acquaintance, moral concept and values* and *degree of specialisation.* An important item in this context was ways to get to know a potential partner. Specific mention was made both of the professional and private level. One suggestion was to jointly experience an extreme situation in order to get to know each other better.

*P19: […] and of course to look at the partner, also in extreme situations. Because it’s always like this: after work or at some event everybody is mostly relaxed and happy. But if the worst comes to the worst and things are very busy, in such extreme situations their true colours will show […].*

Deciding to cooperate with a former trainee and to make use of probation time was generally favoured.

*NP10: […] and I notice that the best and most secure collaborations are the ones, when junior doctors, who have been working in the practice join in. [...]if somebody had worked in the practice for some time already, for their specialisation or as a locum, if they know the practice [...].*

On a personal level, it was essential for physicians to respect each other and each other’s medical approaches and to have a similar attitude regarding patients.

*P4*: *Strong differences in moral concepts and values lead to failure, that is quite simple.*

When choosing a partner, some were convinced that the degree of specialisation also influences success, because if each partner has different expertise, it can lower the potential for conflict in terms of reducing the opportunities for comparison among partners.

*NP8: On the other hand, as soon as you are more specialized in a group practice or an association, it reduces comparability; and the less comparability the less trouble develops […].*

### Practice management

The participants recognised practice management as a fundamental factor that can determine success or failure. Sub-themes that emerged were *general organisation, staff, distribution of non-clinical tasks and consulting services.*

They emphasized the importance of organization in general, which implied tasks like arranging team meetings on a regular basis and sharing administrative duties and clinical work. Participants commented that professional organisation can influence collaboration positively, because subjective perceptions do not carry so much weight.

*NP1: But they [reasons for failure] are based on, I don’t like something, or someone is working less or more or the other way around, or I make proportionally less money and so on. But this is always subjective and the more professional the level, meaning the more people work together, the more intense the organisational structure, maybe with a manager or as a limited company, the less relevant these elements become, and fewer will fail. So, I think if you want to build a long-lasting arrangement, you have to try to raise the professional level.*

Within the scope of practice management, dealing with staff and the leadership role were seen as important issues that have a high potential for conflict.

*P4: We treat staff very differently, which makes them insecure. This was one condition that led to the failure of our practice.*

Participants also felt that it was unfavourable to have too much staff turnover. On the other hand, they emphasized the benefits of having trainees at a practice, for instance in the form of medical assistants.

*P18: […] we have medical assistants that have been working for us for many years... Most of them were trained by us [...]*

A fair distribution of non-clinical duties among the partners was considered necessary. The latter included administrative tasks, billing and, where appropriate, the delegation of work to consultants. Respondents from both groups underlined the importance of a clear division of responsibilities to avoid imbalances in workload, which, according to the participants, are often the cause of dissatisfaction and conflict. This applies above all to administrative tasks that arise in addition to patient care.

*NP11: We create something for each practice that we call the “organisational chart” […] This means that a partner who is good with numbers is responsible for controlling and finances but informs the others and brings them in. And the same* vice versa*. Someone else is responsible for staff […] and as a result, decisions are made and things don’t just happen […*].

A positive side effect mentioned was that distribution of these tasks reduces the administrative burden for the doctors and enables them to focus more on patient care.

*P24: […] what is also important […] is to have optimal administration, so the doctors can focus more on the medical tasks and work and economic and administrative things are outsourced.*

Delegation of non-clinical tasks was also perceived as an important aspect of practice management, especially in the context of accepting support from consultant services. Participants felt that group practices would benefit from a tax consultant to advise them on the billing and help them organise the allocation of profits. Just as a medical lawyer should help with setting up a collaboration contract.

*P 23: I would say spend some money and get professional advice. That means having someone who is experienced with medical law, a solicitor who can draft a group practice contract, just like a tax consultant who can give advice to both parties […].*

### Preparation for group practice

Participants saw many important aspects in connection with *preparation for group practice* itself*.* Sub-themes identified were *contractual protection*, *lack of work experience*, *time management a*nd *use of consulting services.*

Lack of preparation seemed to be a significant reason for failure. In particular, many referred to the contractual arrangements. Several expressed concerns that doctors show little interest in collaboration contracts and seemed to rely on the fact that as two doctors they would get along well with each other. They insist that mutual aims should be fixed in writing and suggested incorporating built in break points to release the pressure on the partners to make the practice succeed.

*P4: […] define agreed target and, this is totally necessary, to make contracts […].*

Participants also identified the lack of work experience in an office-based practice as a negative aspect.

*P19: […] what I would advise someone above all else is first of all, to work in a private practice, even if only for some hours or weeks, just to see the work routine. […] Because private practice is very different from working in a hospital […].*

Another negatively perceived effect was poor time management. In particular time pressure during the establishment and hasty collaborations.

*NP9: […] it’s always successful if they start planning in time, sensible planning, this is a common issue, even overnight, because there is a lot of time pressure.*

Overlapping with communication category, participants emphasized that it was important to take as much time as needed to discuss all aims in advance. Both groups reported that doctors seemed to be more naive about getting into group collaboration than entrepreneurs from other professions. They had also observed collaboration between doctors based purely on trends, which is a very unfavourable basis for inter-physician collaboration.

*NP3: […] especially recently there are young doctors who read in the press, in a medical journal or on the Internet, that collaboration is important and the way to work in the future. They randomly start a group practice because the world seems to be loudly demanding it.*

Furthermore, participants did not understand why doctors would not make full use of existing support services. The KV can provide useful information about possible forms of establishment and billing procedures. Some saw a problem in particular with the predominantly negative attitude towards KV.

*P21: […] many see our professional body, the Association of Statutory Health Insurance Physicians almost as an enemy if you want to put it that way. This is very foolish because it’s a very competent, powerful, and supportive organisation […].*

### Financial aspects

In the view of both participant groups, failure and success often depends on financial aspects. *The allocation of profits, accounting* and *disappointed synergies* were classified as sub-sections. Many mentioned that disputes arising over money are especially tricky and difficult to overcome. Bankruptcy was explained as a theoretical scenario but was very rarely seen as a practical reason for failure.

*NP1: [...] financial factors are good across the board [...] but that normally works well for physicians, it’s not the problem that they get drawn into great financial difficulties.*

In particular, *the allocation of profits* is a conflict-ridden issue. Both groups reported that the partners often felt that they were not being adequately paid and that they felt wronged. Participants felt that transparency in this process is of great importance and also the best way to prevent conflicts in this area. However, the participants mentioned that it is very difficult to list all activities in a practice in a concrete remuneration system, as there are many activities which are difficult or impossible to calculate or compare, but constitute a lot of work.

*P23: I think most collaborations fail because of the question of who gets how much money when and for what?*

In the context of remuneration, the pace of work, compensation for private patients as well as changed behaviour regarding private spending of the physicians were mentioned as common sources for conflicts. It can lead to an imbalance if the duration of consultations varies significantly between the physicians due to differences in working pace. Patients only want to see the doctor who takes more time, while the other colleague feels pressured to “clear” the waiting room.

*P4: In our situation, in particular the time we spend with the patients. One of us takes relatively long, with long consultations for the patients, the other one is trying to get more patients through, to keep the practice running at a good rate.*

Several interviewees perceived an adjusted compensation model for new partners who entered collaboration as advantageous to reduce conflicts about the allocations of profits due to differences in working speeds.

With regard to accounting, the participants found it helpful to outsource the billing of medical services, especially the use of a control system that detects problems. In addition, they pointed out the disadvantages of quarterly billing, which entails the risk of unforeseeable developments.

*NP2: They have an early warning system that realizes very quickly if something is going on. [...] the smaller [practices] send their billing to the KV every quarter and wait to see what will happen instead of having done it in an organized fashion [...]*

Participants believed that the way in which bank accounts are used could provide important information about whether group practices work well. It makes a difference whether it is a shared account and who has access to it. Several participants described the irregular withdrawal of money as a warning signal that should alert the partners.

*NP3: […] but if the withdrawals of the shared bank account become irregular, become more frequent, if they exceed the arranged agreements. Those are warning signals […].*

Another explanation for failure in terms of financial aspects was that physicians became disappointed when the synergies they had hoped for did not manifest in the collaboration.

*NP3: […] physicians expect that if they enter a collaboration, that they can basically cut their expenses in half. But it doesn’t work like that, and sometimes if expectations are too high, that can be a reason for failure, too.*

### Lack of entrepreneurial skills

Many participants saw the perceived lack of entrepreneurial skills among physicians as a determinant for failure. Sub-themes in this category include *lacking preparation for self-employment, economic complexity, lack of comprehension o the economics* and *practice concept.* They argued that doctors were not prepared for self-employment and that this was partly a problem of undergraduate training but also a deficit of postgraduate training.

*P21: A lot of naivety. Physicians start a private practice without any preparation. In terms of human factors, economic or any other form of organisational preparation. They just do it.*

They felt that many doctors underestimated the complexity of having their own practice and have little understanding of the economics.

*NP13: What is very important for me is entrepreneurial awareness. Many doctors stumble into a collaboration without realising that they have equal partners, that they share an entrepreneurial position. They underestimate the decisions they have to make in important business matters, in contractual matters.*

The participants stressed the difference between working in private practice and in a hospital, especially the fact that physicians working in hospitals are salaried and do not have to deal with business matters such as billing or staff.

*NP2: […] they don’t talk about where they are going with the practice, how to handle self-payers, how much money do I require for my personal needs, how should we handle staff issues […] They don’t know this, if they have only worked in a hospital.*

In this context, it was mentioned that having a well thought-out practice concept was very rare yet essential. The specific element here was not so much a finance plan as an agreement between the partners of the practice goals, e.g. profit orientation, investment and interest in developments.

*NP11: I think one of the main reasons is a deficit that almost every practice has, that there is no coordinated cooperative concept among the partners. […] I believe that numbers are easy, and every economist can illustrate numbers and build a finance plan. The more difficult part is to agree on ideas for a concept like: Where do I want to go with my practice? How does it fit? What does my partner want? What do we want to offer in medical terms? And all these things […].*

*P23: I think if someone is very profit-oriented and the other one is not, that can be a reason for them drifting apart. But also professional issues or investment decisions.*

*NP15: […] or if there are different ideas about how modern a practice should be, when discussing purchasing technical equipment or furniture.*

## Discussion

### Principal findings

We identified several determinants that may influence success or failure of group practices, some of them modifiable.

These are communication behaviour, practice management, knowledge about preparing for group practice, financial aspects and a lack of entrepreneurial skills.

Physician and non-physician participants both agreed with the main themes regarding the collaboration partner, practice management, preparation for collaboration and the lack of entrepreneurial skills.

### References to previous research

The majority of results concerning determinants for failure are mirrored in a French study [28]. Unlike in this study, insufficient income was not presented a reason to leave a group practice and the lack of entrepreneurial skills was not characterised explicitly. The sample of the French study consisted only of GPs that had experienced a partnership failing, while we also included specialists of other fields as well as participants with positive experiences. However, the similarity of results might imply that detected determinants could apply in the failure of working collaboratively in group practices on an international level as well.

Although communication skills in clinical settings have been identified as one of the basic competencies for physicians, for example in educational frameworks such as CanMEDS and the German National Competency-based Learning Objectives Catalogue (NKLM), there is still little training in peer to peer communication [38, 39] and research on medical communication mostly focuses on doctor-patient communication or the role of inter-professional communication in terms of patient safety or patient satisfaction [40–42]. It seems that the role of communication for successful inter-physician collaboration has not been well addressed to date. However, for example in the case of physiotherapists, communication among each other has been highlighted as a key competency in intra-professional practice [43]. The improvement of inter- and intra-collegial communication through training was described previously [44].

In line with our findings, Norwegian GPs felt the need for formal training for leadership roles and perceived a conflict between their roles as leader and clinician [45]. This might also be true for German physicians, since there is almost no training in leadership to date and the role conflict might explain the physicians’ inexperience regarding handling staff and other management tasks that the participants described.

Participants blamed the lack of economic understanding as a shortcoming in under- and postgraduate training. The absence of formal management courses in postgraduate training has been addressed in the Netherlands and mandatory medical management training was recommended after a successful pilot in practice management training [46]. The perceived economic risk in private practice, uncovered in a German survey of prospective physicians has already initiated reflections about management skills among medical students [25]. With regard to future education, business and financial management were determined as high priority content and a simulation game as educational strategy has proven effective [19, 47]. In order to integrate practice management in postgraduate training in Germany, some regional KVs already offer special workshops in management training for practice establishment [48]. Special competence centres have been set up to optimize postgraduate training in general practice to improve and centralize training. Several of these centres have recently integrated management and economy modules into their curriculum [49].

### Strength and weaknesses of the study

Ours is one of very few studies that address the success or failure of inter-physician collaboration in group practices. The study benefits from the inclusion of different professions, different medical specialisations and the qualitative design to examine details in depth. Nonetheless, it should be borne in mind that there may be differences in the collaboration of different sub-specialisations, as there are different basic requirements.

Although it was a consideration that telephone interview would detect fewer nuances due to the lack of non-verbal responses, we nevertheless opted for this interview form in order to involve participants from different regions and facilitate participation, as most interviews were conducted by appointment. However, as the interviews were rather short, it is possible that answers were affected by time pressure or distractions. With consideration to quality criteria for qualitative research, we sought to gain transparency by documenting our approach rigorously and carrying out data analysis as a group [37].

The majority of the sample was male. This could represent a limitation, as the views of women were not caught in a likewise manner. This could matter especially in terms of gender distribution in medicine, which is increasingly skewed towards women [50].

A selection bias of the results due to highly motivated or frustrated participants cannot be ruled out completely. It is possibly that individuals interested or positively inclined towards our research were more likely to participate or by contrast that that discontent individuals may have shown more interest. Participants also may have been more aware of the pitfalls of inter-physician collaboration than non-participants. We also have to take recall bias into account, as participants reported on former experiences.

We should also keep in mind that the organisation of health care systems and legal frameworks for collaboration in the medical field vary from country to country. Physicians in other countries might therefore have different experiences regarding reasons for failure or success of working collaboratively in group practices.

As a qualitative approach, this study serves the purpose of generating hypotheses and its results should therefore be generalized with caution.

Future research concerning inter-physician collaboration should also incorporate internal group practice factors like composition and size of the team, existence and number of administrative staff and external assistance by consultants.

### Deduction/ hypothesis

The first implication is that among the versatile determinants we identified, there are several themes that might be improved by broadening specific training for physicians, especially beyond the clinical arena, focusing more on communication in general and, in particular, with colleagues of different hierarchy levels and also to foster social competences like teamwork, reliability and respectful contact with peers.

Therefore learning and practicing peer to peer communication is essential for successful inter- physician communication too. Peer to peer communication training should be incorporated into postgraduate training or offered as workshops for practice teams, which might enhance communication skills for working collaboratively in group practices as well. It was stated that physicians had mostly learned collaboration skills from role-models. The necessity to investigate new ways to teach and train collaboration and conflict negotiation with physicians has been pointed out previously [51].

A higher level of expertise in preparing for group practice seems necessary as well. In Germany, many regional KVs already offer additional information, such as a road map to setting up practice in which useful information for new physicians in private practice is compiled [52, 53]. There are also mentor programs as offered by the competence centres that connect experienced office-based physicians with prospective ones [49]. Given our findings, the existing offers need to be strengthened and improved.

Education in practice management, financial aspects in group practice and entrepreneurial skills are competencies that are missing in the current medical curriculum but seem to be crucial for working successfully in group practices [54]. Thus, education in this area is urgently needed in undergraduate training.

In Germany the legal allowances for specialist and GP practices are similar. This suggests that our findings can also be applied beyond GP group practices in Germany. Considering the agreement with earlier studies from abroad, it suggest that to some extend this is also true on an international level.

## Conclusion

In order to positively influence future collaboration, it is important to impart management and communication skills as well as a basic understanding of economics and to expand knowledge about collaboration. The development, implementation and identification of the optimal time during training for such education strategies should be the topic of future research.

## Data Availability

The datasets used and/or analysed in this study are available from the corresponding author on reasonable request. Datasets are in German.

## Abbreviations

GP(s): General Practitioner(s)
KV (Kassenärztliche Vereinigung): Association of Statutory Health Insurance Physicians
NP: Non-physician group
OECD: Organisation for Economic Cooperation and Development
P: Physician group
